# Pain management in hemophilia: expert recommendations

**DOI:** 10.1007/s00508-020-01798-4

**Published:** 2021-03-04

**Authors:** Waltraud Stromer, Ingrid Pabinger, Cihan Ay, Richard Crevenna, Josef Donnerer, Clemens Feistritzer, Sophie Hemberger, Rudolf Likar, Florian Sevelda, Katharina Thom, Barbara Wagner, Werner Streif

**Affiliations:** 1 Department for Anaesthesia and General Intensive Care, Horn State Hospital, Spitalgasse 10, 3580 Horn, Austria; 2 Clinical Division of Haematology and Haemostaseology, Department of Medicine I, Medical University of Vienna/Vienna General Hospital, Vienna, Austria; 3 University Department of Physical Medicine, Rehabilitation and Occupational Medicine, Medical University of Vienna/Vienna General Hospital, Vienna, Austria; 4 Medical Faculty, Johannes Kepler University, Linz, Austria; 5 University Hospital for Internal Medicine V/Haematology and Oncology, Medical University of Innsbruck, Innsbruck, Austria; 6 University Department of Paediatric and Adolescent Medicine, Medical University of Vienna/Vienna General Hospital, Vienna, Austria; 7 Department for Anaesthesia and Intensive Care, State Hospital Klagenfurt am Woerthersee, Klagenfurt, Austria; 8 University Department of Orthopaedics and Trauma Surgery, Medical University of Vienna/Vienna General Hospital, Vienna, Austria; 9 Department of Pediatrics, Medical University of Innsbruck, Innrain 52, 6020 Innsbruck, Austria; 10 Wolfsberg State Hospital, Wolfsberg, Austria; 11 Palliative Care, Sigmund Freud University Vienna, Klagenfurt, Austria

**Keywords:** Hemophilia, Factor concentrate, Pain therapy, Joint bleeds, Pain chronification

## Abstract

**Introduction:**

As a typical consequence of bleeding into muscles and joints, patients with severe hemophilia suffer from acute and chronic pain. In spite of its high prevalence, pain in this patient group is not always sufficiently considered or treated in an effective manner.

**Aim:**

The recommendations presented in this paper address possible improvements in pain management in hemophilia patients and particularities that have to be taken into account in this patient group.

**Method:**

The manifold aspects of pain management in hemophilia patients were discussed within the framework of an expert meeting. Based on the available literature and the experts’ clinical experience, the participants developed a set of recommendations presented in this paper.

**Results:**

Pain management in patients with hemophilia is often insufficient, a fact that not only influences the patients’ quality of life but also implies the risk of difficult to manage chronic pain. Both the prevalent polypharmacy (due to comorbidities) as well as the underlying disease itself present special challenges to pain therapy in this patient group. The present review and recommendations are intended to support medical professionals in recognising the risks of pain chronicity, applying basic principles of multimodal pain therapy, including the options of psychological intervention and modalities of physical medicine in therapy concepts, and reaching a comprehensive understanding of the range of analgesic options available.

## Background and goal

Nowadays, life expectancy in patients with hemophilia A or B is similar to healthy populations, provided the appropriate therapy is available; however, severe forms of this coagulation disorder with acute and chronic pain due to bleeds in joints and tissue structures pose a significant problem. Modern therapy options have helped reduce the prevalence of these bleeds, but even with consistent therapy, bleeds cannot be completely avoided. Competent management of the bleeds as well as targeted pain therapy are required.

While general guidelines for the management of hemophilia [1, 2] are available, there are no evidence-based guidelines or detailed algorithms that specifically deal with pain management in hemophilia patients. As far as pain management in children and adolescents with hemophilia is concerned, interdisciplinary treatment recommendations were published in 2018 [3]. An interdisciplinary group of experts (pain therapy, haematology and haemostasis, orthopaedics, paediatrics, physical medicine, psychology) has now developed recommendations for pain therapy in hemophilia patients which we present in this paper.

## General remarks on hemophilia

Hemophilia A, which is caused by a deficiency of clotting factor VIII, has an incidence of 1:10,000 making it a relatively common coagulation disorder. With an incidence of 1:50,000, hemophilia B, which is characterised by a deficiency in factor IX, is less common. Clinical bleeding tendency correlates with factor activity. Patients with severe hemophilia require lifelong prophylactic treatment with factor concentrates to prevent bleeding [4]. This entails the regular administration of factor concentrates. Untreated and recurring bleeds lead to pain, functional restrictions, and disability [1, 5].

## Prevalence of pain and insufficient treatment

For patients with hemophilia, pain is a lifelong issue [6]. From their early years, patients often experience acute and chronic pain, particularly due to joint bleeds or joint degeneration, but also due to therapeutic interventions. A patient survey carried out in Germany in 2013 illustrates how pain affects the patients’ quality of life [7]. Of the patients surveyed 86% suffered at least occasionally from hemophilia-related pain. Of the respondents, 92% listed joint pain as the most frequent type of pain. In children and adolescents (aged 0–17 years) 66% of the hemophilia patients experienced pain. As hemophilia patients grow older, they seek medical assistance due to pain with increasing frequency. Of those looking for assistance, 40% contact a hemophilia specialist, 32% a general practitioner (GP), while 9% seek out the help of an orthopaedist. For specialised pain treatment, 46% of respondents were referred to an orthopaedist and 31% to a physiotherapist. In order to improve pain therapy, consulting a pain expert should not be seen as a last resort, but should rather take place much sooner, particularly in order to prevent pain chronicity.

Causes for insufficient pain management in hemophilia patients can be manifold. In many cases, hemophilia-related pain is underestimated [8], which leads to an insufficient utilisation of pain therapy options. Uncertainty about the choice of therapy in view of the coagulation disorder may also lead to inadequate pain therapy.

## Pain in hemophilia patients

More than 80% of all bleeds affect joints, of which the most frequent is the knee, followed by ankle, elbow, and wrist [7]. Consequently, these joints are the most frequent locations of acute and chronic pain.

Acute pain may be caused by bleeds, chronic pain by long-term degeneration such as arthropathy.

Joint bleeds are provoked by the low levels of coagulation factor and are associated with inflammation and pain. In case of recurring bleeds, the angiogenesis of blood vessels in the synovia further increases bleeding propensity. Chronic joint effusion then leads to the formation of a pannus, which affects functionality, causes joint degeneration and pain [8, 9].

Surveys on chronic pain in connection with hemophilia show that 32–50% of hemophilia patients suffer from arthropathy-related pain [10]. This type of chronic joint pain leads to physical inactivity and therefore also an increased risk of cardiovascular events and has a negative influence on psychological health. It precipitates social isolation, limits the ability to work, and often gives rise to the use of potentially addictive substances.

Larger bleeds in the muscle tissue can, if untreated, lead to increased pressure and calcification, causing damage to nearby nerves and blood vessels, and therefore also leading to pain and limited range of movement, muscle atrophy, and paralysis.

Aside from disease-specific pain, hemophilia patients can also suffer from all other common types of pain including headaches, painful gastrointestinal symptoms, and postoperative pain after surgical interventions, which all need to be appropriately treated while taking both the underlying disease and the resulting contraindications into account.

## Pain chronification

For patients with hemophilia acute pain is usually a warning signal for acute bleeds in joints or muscles, or the result of a traumatic event.

Insufficient or inadequate analgesic control of acute pain leads to a sensitisation of peripheral as well as central neurons; noxious, painful stimuli are perceived as increasingly strong (hyperalgesia).

Additionally, susceptibility to non-noxious or non-painful stimuli increases (allodynia) [11, 12].

Appropriate pain therapy at the time of the initial pain stimulus can mitigate, possibly even prevent, the negative long-term effects [13]. In order to avoid pain chronification, treatment must anticipate acute pain by providing continuous pain management [14].

The goals of acute pain therapy are:

- the mitigation or avoidance of nociceptive input from primary afferents,
- the blocking or mitigation of inflammation, and
- the inhibition of ectopic activity in neural structures.

Aside from inadequate pain treatment, factors like depression, distress, a tendency to catastrophize the pain sensation, feelings of helplessness or hopelessness, fear-avoidance behaviour, suppression of even the thought of pain, or a tendency to somaticize are among the risk factors for pain chronification [15].

## Measuring and documenting pain

In order to enable adequate pain treatment, it is necessary to effectively and systematically measure and evaluate the pain [16] in order to optimise therapy; however, the reality of treatment does not always seem to reflect these insights. The European Hemophilia Therapy Standardisation Board [10] has analysed 22 European hemophilia centres in 14 European countries with respect to their pain management. The results showed great disparity: only eight hemophilia centres had standardised pain measurement with scales and internal guidelines for pain treatment.

In general, hemophilia patients should use self-reporting tools; specific pain questionnaires for self-assessment have proven useful when it comes to targeted preparation for the medical consultation [16].

Pain scales are a convenient option for pain measurement. They enable the structured monitoring of the course of the condition and therefore also of the effectiveness of pain therapy. The visual analogue scale (VAS) or the numeric rating scale (NRS) have proven useful for quantification [17]. Due to possible misleading conclusions, the still widely used “Smiley Scale” is not recommended. The faces pain scale [18] is used for self-assessment of pain severity, a useful instrument, inter alia, for foreign language patients.

In order to take the particularities of pain in hemophilia patients into account (such as the simultaneous occurrence of acute and chronic pain or the prevalence of multiple pain locations), specific tools for pain evaluation in hemophilia have been developed. The Multidimensional Hemophilia Pain Questionnaire (MHPQ), which distinguishes between acute and chronic pain and assesses a number of relevant pain dimensions, is supported by validation data [19].

In persons with cognitive impairments with a Mini-Mental State Exam (MMSE) score of <15, face pain scales should be used as primary tools. For patients with advanced dementia, external pain assessment tools, such as DOLOPLUS‑2, DOLOPLUS-2-SHORT, BESD (*Beurteilung von Schmerzen bei Demenz*) or PAINAD (pain assessment in advanced dementia) are available. Further tools include EPCA (*L’échelle Comportementale pour Personnes Agées*) and BISAD [20–24].

In this context, the validation and definition of intervention limits are of the utmost importance to ensure that pain measurement actually leads to therapeutic results. These limits are pain values ≥3/5 (rest pain/movement-induced pain) in the context of surgery and pain values ≥3/4 (rest pain/maximum pain) in the field of conservative therapies.

## Pain therapy in hemophilia patients

### Particularities and special challenges

Treatment of pain in hemophilia patients generally follows the same criteria and recommendations as with other pain patients; however, there are some particularities that need to be taken into account.

Hemophilia is characterised by bleeding tendency. The choice of analgesics must therefore take into account whether or not a specific substance inhibits coagulation and/or impairs platelet aggregation.

This goes in particular for acetylsalicylic acid (ASA) but also for other non-steroidal anti-inflammatory drugs (NSAIDs) that have an effect on cyclooxygenase (COX)-1. In the case of acute bleeding, the immediate administration of factor concentrates for patients with severe hemophilia is indicated; however, aside from managing the bleeding, adequate pain management must be an immediate concern as well, especially since treatment of acute pain is paramount for chronification prophylaxis (see also Section “Pain chronification”).

### General principles of pain therapy

In persons with hemophilia, both therapy of acute and of chronic pain are of high relevance.

The major aims guiding acute pain management are to provide a treatment that reduces pain quickly and effectively with a minimum of side effects and permitting patients to maintain function, while also preventing the development of chronic pain. Acute pain treatment needs to be implemented whenever a threshold of NRS >3 (at rest) | NRS >4 (in motion) is reached; strategies of pharmacological interventions follow a mechanism-based medication concept.

As for chronic pain treatment, multimodal and multiprofessional therapy strategies should be chosen for hemophilia patients. The cornerstones include factor substitution, maintaining adequate coagulation, systemic pain therapy, exercise therapy and physical medicine, ergotherapy, psychotherapeutic/psychological care, and complementary measures, such as acupuncture and neural therapy.

The goal of multimodal pain therapy is to improve analgesic quality by combining a variety of interventions in the best possible way, to avoid painful experiences and side effects, to minimise physiological stress responses to pain, and to boost recovery.

### Pain therapy

#### Psychological pain interventions

The significant psychological strain that comes with chronic pain often receives too little attention. In patients with a chronic illness, the likelihood of developing mental impairments is increased twofold or threefold [25], a fact that must be considered when dealing with hemophilia patients. Possible comorbidities include depressive episodes, anxiety disorders, and personality disorders. The extent of these illnesses is decisive when it comes to resources and deficits with respect to pain processing and coping.

Not all hemophilia patients who suffer from chronic pain require psychological intervention. Indications for such an intervention, however, are dysfunctional pain experience and behaviour, insufficient stress management, psychosocial conflicts, issues at work, compliance issues, substance abuse, or psychiatric comorbidities.

The goals of psychological interventions include the patient dealing with the chronic illness, defining realistic goals, reducing patterns of fear and avoidance, creating strategies for coping with pain and stress as well as developing adequate pain communication and problem-solving approaches. This helps improve compliance and quality of life.

The first steps before any psychological treatment include in-depth anamnesis and exploration, often followed by psychological diagnosis based on available standards of diagnostics [26]. The next step would ideally be interdisciplinary therapy planning, something that is usually only possible in large institutes. Examples of possible psychological interventions include:

- Psychoeducation
- Relaxation techniques
- Biofeedback
- Hypnotic and non-hypnotic imaginative processes
- Mindfulness exercises
- Operant processes
- Cognitive behavioural approaches

#### Modalities of physical medicine

As a component of multimodal pain management [27], physical medicine and rehabilitation play an important role by functioning as complementary treatments for acute pain and by preventing and treating chronic hemophilic arthropathy and the pain this causes. In collaboration with other disciplines, physical medicine thus contributes to improving or maintaining functionality.

As part of the overall treatment of hemophilia, exercise therapy plays an important role because many patients suffer from deficits in several basic motor functions: strength (especially in the extremities and back muscles) [28–32], coordination/balance/proprioception [31, 33–36], flexibility [33, 36], and endurance performance [30, 36].

Chronic hemophilic arthropathy is associated with pain, strength deficits, muscle atrophy, contractures with inhibited range of motion as well as impaired intermuscular and intramuscular coordination. Hemophilic arthropathy leads to joint instability and angular deformities and may finally result in joint ankyloses and disability [1, 37–40].

Consequently, multimodal exercise is recommended to improve coordination, flexibility, range of motion, strength, general functionality and general fitness [1, 41–43]. A main goal is improved joint stability. Central and peripheral pain-reducing mechanisms of exercise are being discussed [44–46].

Resistance exercise in combination with coordination and endurance training can improve joint stability and reduce the risk of injury, falls and bleeding [1, 10, 37, 39, 41, 43, 47]. Adequate and individually adapted resistance exercise improves control over maximum degree joint movement and can reduce synovial impingement with associated bleeding or synovitis, while also promoting good bone density and possibly reducing pain in cases of arthrosis [8, 41, 48]. Aerobic exercise can reduce the risk of obesity and metabolic and cardiovascular diseases [43, 48].

Aquatic therapy is an important therapy option for patients with hemophilia since it seems to reduce pain more effectively than land-based exercise, at least in adult patients [45]. Aquatic therapy also leads to oedema reduction and a reduction of muscle spasms. It helps maintain range of motion, while improving balance, coordination, strength, and posture [8, 49, 50].

Since pain leads to a change in joint function and to impaired motion sequence as well as to reduced therapeutic and exercise success, adequate analgesic treatment is essential before engaging in physical activity [51]. At the same time sufficient factor supply in line with recommendations and guidelines must be ensured [1, 42, 43]. Exercise should be adapted to the patient’s needs, deficits, level of fitness and joint status. It should be prescribed accompanied and increased by musculoskeletal specialists with experience in hemophilia [1, 42, 43].

Physical medicine also has a prophylactic potential. Functional disorders caused by inflammatory processes often precede structural changes in joints and thus increase the risk of joint bleeds [52, 53]. Different examination methods can be used in order to catch structural or functional changes in joints early on, the basis for this being regular (depending on age and symptoms) and in-depth physical examinations [53]. The hemophilia joint health score can be used for a thorough musculoskeletal assessment that includes the systematic detection of structural and functional arthropathic changes [54]. In so far as they are available, further examinations, such as surface electromyography (EMG) and gait analysis can be used in order to detect possible individual functional deficits, functional disorders of the muscles, inappropriate weightbearing, and impaired motion sequence [52, 54]. Diagnostic imaging procedures that are useful for detecting osteochondral defects and synovitis include magnetic resonance imaging (MRI, gold standard) in particular, as well as ultrasound [52, 54]. The joints that are most commonly affected by hemophilia can be screened for early arthropathic changes using ultrasound (e.g., HEAD-US protocol) [54, 55]. For optimal adaptation of exercise therapy to a patient it is important to measure their physical fitness, e.g., using a dynamometer or ergometer. By way of early diagnosis of impairments or deterioration, adequate treatment can be initiated early on, thereby avoiding or reducing joint damage and the associated pain.

For patients with limited access to exercise facilities, home training programmes that are adapted to the disease and made available through online videos are an option. Specialist approval that also ensures adequate factor therapy and, ideally, constant supervision by musculoskeletal specialists are important [56].

##### Electrotherapy

Low-frequency electrotherapy: transcutaneous electrical nerve stimulation (TENS) is used as an analgesic option in case of acute haemarthrosis and chronic pain [49, 57, 58]. Iontophoresis with local NSAIDs stimulates circulation, provides a trophic stimulus and has an anti-inflammatory and analgesic effect [49, 51]. Neuromuscular electrical stimulation (NEMS) can increase muscle thickness and strength, as was shown in studies on the biceps and quadriceps muscles [59, 60]. Diadynamic currents (CP according to Bernard) [39], Trabert current or high voltages are also used for analgesic therapy in people with hemophilia.

Medium-frequency interferential current has an oedema and haematoma-absorbing and analgesic effect [49].

When prescribing electrotherapy (just like with any other physical therapy option), general contraindications have to be taken into account. In children and adolescents, there is a relative contraindication in the area of the epiphyseal plate. Just like with any other measure that increases circulation, using it on areas with an active or potential tissue bleed must be preceded by ensuring appropriate factor protection since most modes of electrotherapy stimulate the circulation locally [61].

##### Ultrasound

In cases where heat generation should be avoided, pulsed ultrasound can be used [57], although evidence is not very strong. The analgesic effect can be improved through phonophoresis, whereby a medical gel with anti-inflammatory and analgesic substances is applied. Ultrasound is particularly effective when it comes to resolving adhesions caused by inflammation and breaking up fibrosis and scar tissue. It positively affects tissue repair and inflammatory processes [51, 52, 57].

##### Massage

Massage techniques can also be indicated for analgesic purposes in patients with hemophilia. Examples include lymphatic drainage (reduces swelling), classical massage (reduces muscle tone, mechanically loosens fibrosis in the connective tissue, prevents adhesions when used subacutely after muscle bleeds) or Terrier massage (indicated in cases of increased muscle tone and limited mobility) [49, 51, 52]. For intense treatment, sufficient factor protection is important.

##### Thermotherapy

During acute phases, the RICE principle (*R*est, *I*ce, *C*ompression, *E*levation) is frequently used to treat pain [1, 8, 62]. In cases of acute joint and muscle bleeds, cryotherapy reduces pain and hypoxic damage, and improves ischemic tolerance in the affected area [63]; however, due to possible negative effects on platelet function and clotting factor activity, the literature reflects some controversy in this area [64]. Cooling is recommended for a maximum of 15–20 min at a minimum of 2‑h intervals. The ice should be wrapped in a dry cloth to avoid direct skin contact [1, 62, 63].

Heat treatment for muscles increases circulation and leads to a reduction in muscle tone; however, heat treatment can lead to increased muscle spindle activity and can consequently lead to reflectory muscle tension after the treatment [51]; factor protection is indicated.

##### Other treatment options

Relaxation techniques like biofeedback, Jacobson’s relaxation technique or mental training as well as proprioceptive neuromuscular facilitation (PNF) can lead to improved body awareness [49, 52].

Acupuncture can also be an effective way of treating chronic pain in hemophilia patients [7, 61, 62], although available publications report about a limited number of patients.

Other good therapeutic options for pain reduction and improvement of functional parameters include laser and pulsed electromagnetic field (PEMF) therapy, which was demonstrated for children in two studies [65, 66].

Depending on the level of physical limitation, orthoses, assistive devices, and insoles may be indicated [8].

#### Pharmacological pain therapy

##### Basics of pharmacological pain therapy

Central elements of successful pharmacological pain therapy include:

- classification of the underlying pain mechanism (nociceptive, inflammatory, neuropathic or nociplastic pain)
- assessment of polypharmacy
- consideration of organ insufficiencies and contraindications
- regular monitoring of therapeutic response and possible adverse effects

In the spirit of a multimodal process, pharmacological pain therapy should always combine a variety of substances in order to try to maximise the analgesic effect. Hemophilia patients are often insecure concerning the pain medication they are allowed to use. Consequently, patient education on contraindicated and suitable substances is of the utmost importance. It is also crucial to bear in mind not just the individual substances’ effects on coagulation but any effect an interaction of different substances might have.

When choosing suitable substances, it is important to evaluate the risk-benefit ratio. The therapy should not just focus on pain intensity but the underlying pain mechanisms:

- Nociceptive pain is caused by a stimulation of peripheral nociceptors and transmission of the pain stimulus along sensitive nerve fibres. Pain in muscles, ligaments, or joints falls into this category. Nociceptive somatic pain is localised and after consideration of possible contraindications and restrictions on use, it is typically treated with nonsteroidal anti-inflammatory drugs (NSAIDs), selective cyclooxygenase‑2 inhibitors (coxibs), dipyrone (metamizole), and paracetamol; this type of pain responds well to opioids, too. In cases of nociceptive visceral pain, dipyrone is a good option; opioids and co-analgesics are also used.
- Neuropathic pain is caused by an irritation of the peripheral or central nervous system, which may be caused by, for instance, an infiltration or compression of neural structures [67].
- In cases of neuropathic pain anticonvulsant drugs and antidepressants, opioids as well as topical therapies, such as lidocaine and capsaicin have therapeutic significance [68].

In order to avoid or minimise adverse effects, analgesic drugs should be used at the lowest possible dosage and for as short a time as possible, but certainly long enough to sufficiently treat the pain and avoid chronification. Since insufficient pain reduction may lead to patients resorting to over-the-counter (OTC) analgesics, regular therapy monitoring is necessary.

##### Topical analgesics for hemophilia patients

Indications for topical analgesics include musculoskeletal pain, ligament injuries, joint diseases, muscle pain syndrome, and various spine-related pains.

The analgesic effect of topical NSAIDs corresponds to about 50% of the effect reached via systemic administration and entails significantly fewer adverse effects. Clinical evidence for the effectiveness of topical NSAIDs is generally quite convincing, particularly when it comes to acute musculoskeletal pain syndromes [69].

Dermal tolerability of topical NSAIDs is good, although topical ketoprofen can cause photoallergic reactions.

Most topical NSAIDs are available in gel form. In order to optimise percutaneous bioavailability, it is important to be generous in terms of both the amount of gel used and application area [70].

Topical applications of lidocaine and capsaicin are suitable for local, external treatment of neuropathic pain.

##### Non-opioid analgesics

Non-opioid analgesics can be combined in order to improve analgesic efficacy, for instance COX inhibitors with paracetamol (acetaminophen) or dipyrone (metamizole). Due to a possible potentiation of the risk of adverse effects, different COX inhibitors, however, should not be combined.

##### Nonsteroidal anti-inflammatory drugs and coxibs

Both NSAIDs and selective COX‑2 inhibitors (coxibs), target the cyclooxygenase enzyme and have analgesic, antipyretic, and anti-inflammatory effects [71, 72].

There are two isoenzymes: COX‑1 and COX‑2 [72]. COX‑1 protects the stomach lining, regulates renal perfusion and induces platelet aggregation via the production of thromboxane A2 in thrombocytes. In inflammation or swelling the net analgesic effect of this drug class can be superior to that of opioids. This goes for, e.g., naproxen, ibuprofen, mefenamic acid, and diclofenac [73]; however, significant adverse effects are possible.

Among this drug class, acetylsalicylic acid (ASA) causes the most problems; even in small doses (30–50 mg), ASA irreversibly blocks COX‑1 in platelets, thus negatively influencing coagulation [74]. Consequently, ASA is thoroughly unsuited for treating pain in hemophilia patients. This also goes for combination preparations with ASA.

As they have an anti-inflammatory effect and pose a lower risk of gastrointestinal adverse effects COX‑2 inhibitors, such as celecoxib are better suited for pain therapy in hemophilia patients. Etoricoxib was investigated in patients older than 12 years (*n* = 102) and proved to be effective, safe, and well-tolerated when administered to patients with arthropathy [75]. Due to the well-known renal, cardiovascular, and gastrointestinal safety profile, coxibs as well as NSAIDs should only be prescribed in the lowest effective dosage and for as short a time as possible. In cases of an existing cardiovascular comorbidity the COX‑2 inhibition leads to an exponential risk of recurrent cardiovascular events [76, 77]. Severe cardiovascular underlying diseases and cardiac insufficiency are therefore to be regarded as contraindications for COX‑2 inhibitors.

When prescribing NSAIDs special attention should be paid to possible polypharmacy (Table 1). Administering Angiotensin-converting enzyme (ACE) inhibitors alongside NSAIDs can reduce the antihypertensive effect of this substance class and potentiate the risk of renal failure. NSAIDs also increase the risk of bleeding (especially gastrointestinal bleeding) when anticoagulants, corticosteroids, and selective serotonin reuptake inhibitors (SSRIs) are administered simultaneously [78, 79]. The use of antidepressants, antipsychotics, anticonvulsant drugs, and opioids can cause a severe electrolyte imbalance like hyponatremia. Alternatively, non-opioid analgesics such as paracetamol and dipyrone (metamizole) are an option for treatment.

**Table 1:** Summary of selected analgesics interactions

NSAIDs	SSRI, corticosteroids	Increased bleeding risk (especially in the gastrointestinal tract)
	In combination with ACE inhibitors risk of renal failure	Risk of renal failure
	With antidepressants, anticonvulsants, antipsychotics and opioids	Increased electrolyte imbalance like hyponatremia
Paracetamol	With 5‑HT3 antagonists (e.g. ondansetron or tropisetron)	Reduction of analgesic efficacy
Antidepressants	–	Increased incidence of arrhythmia in patients with cardiac impairments
Gabapentin	With antacids	Reduced resorption (at least 2‑h intervals between doses)
Pregabalin	With oxycodone	Possible impairment of cognitive and gross motor skills

*NSAID* nonsteroidal anti-inflammatory drugs, *SSRI* Selective Serotonin Reuptake Inhibitors

##### Paracetamol

In many cases, paracetamol (acetaminophen) is currently used as first-line therapy for treating pain in hemophilia patients even though no randomised clinical trial data for hemophilia patients are available.

Paracetamol is indicated for mild to moderate levels of pain. It has an antipyretic but no anti-inflammatory effect, and shows some COX‑2 inhibition. The recommended dosage to be administered at any one time is 10–15 mg/kg body weight. When overdosed multiple times paracetamol has a hepatotoxic effect, and so the approved maximum individual dosage of 15 mg/kg body weight or maximum individual dosage of 1 g and maximum daily dosage of 4 g for people weighing >50 kg should never be exceeded [80]. Overdosing can lead to liver failure. Paracetamol does not inhibit platelet aggregation. Due to the peripheral selective COX‑2 inhibition, prolonged paracetamol administration increases the cardiovascular risk. According to available studies, 1g of paracetamol inhibits COX-2 at a rate of about 83% [81]. High doses and prolonged administration can also lead to an increased risk of renal and gastrointestinal complications [82]. In cases of hepatic insufficiency, paracetamol is contraindicated.

When paracetamol is administered together with 5‑HT3 receptor antagonists, the analgesic effect can be reduced (Table 1). Caution should be exercised in cases of alcohol abuse and in combination with CYP2E1-inducing drugs due to the resulting faster metabolisation, which can lead to an increase in hepatotoxicity. In such cases, the dosage of paracetamol must be reduced.

##### Dipyrone (metamizole)

Compared to paracetamol, dipyrone (metamizole) has a relatively high analgesic potency and is especially useful for visceral pain and colic. It has an antipyretic effect and can therefore be used to treat feverish infections, replacing NSAIDs which are problematic when treating hemophilia patients.

Dipyrone has low interaction and adverse effect potential [83, 84]. Dipyrone-induced agranulocytosis is a rare adverse effect in Central Europe; however, when administered over longer periods of time, regular blood cell counts are indicated [85]. Overdosing leads to relatively low renal, hepatic, and gastrointestinal toxicity.

The temporary platelet dysfunction caused by dipyrone (metamizole) has been known for a long time and its clinical relevance is a matter of discussion [84, 86, 87]. Consequently, the use of dipyrone (metamizole) in cases of known platelet dysfunction cannot be advised. In case of hemophilia, factor deficiency is most relevant. Refusal of short-term dipyrone (metamizole) use for the treatment of headaches, colic, pain in the soft tissue or fever is not justifiable. In a postoperative setting and in case of severe trauma, factor substitution and balancing coagulation in hemophilia patients is paramount. If dipyrone (metamizole) is used in such situations, a risk-benefit evaluation is indicated. In cases of renal insufficiency, the dosage of dipyrone (metamizole) should be reduced.

##### Opioid analgesics

In accordance with their analgesic potency, weak opioids (tramadol, nalbuphine) and strong opioids (fentanyl, buprenorphine, morphine, hydromorphone, oxycodone, piritramide) are available.

The use of opioids is always indicated in combination with non-opioids as basic medication. By combining two non-opioids with a different mode of action, e.g., a coxib plus dipyrone (metamizole), the analgesic effect is increased leading to a possible opioid reduction of up to 40% [88].

If non-opioids fail to reach the desired analgesic effect or if pain intensity is high, it is indicated to complement the therapy as soon as possible with opioids during an acute phase. That is also important to avoid chronification mechanisms.

The opioids are first administered intravenously, fractionated and titrated, or in an immediate release form that is given orally. With chronic joint pain, limited mobility, and inappropriate weightbearing, constant analgesic medication using a non-opioid plus extended release opioids may be required.

One advantage of opioids when compared to non-opioid analgesics is the low organ toxicity. The paramount principle when using opioids is start low, go slow: due to initial adverse effects like fatigue and dizziness opioids should first be administered in low doses and an increase in dosage should only be considered while constantly monitoring effects and adverse effects. Other undesired effects when administering opioids during an acute phase can include cardiovascular depression, sedation and the risk of falling that comes with it, pruritus, spasms in the urogenital area/urinary retention, and respiratory depression. The danger of respiratory depression is particularly high when opioids are given intravenously and where either the initial dose was too high, or the dosage was increased too quickly [10]. Antiemetic prophylaxis at the beginning as well as constant obstipation prophylaxis are to be considered obligatory during opioid therapy.

The choice of opioid depends on the pain category, the pain intensity, the pain character and rhythm, possible comorbidities or contraindications, and the receptors involved.

For tramadol a ceiling effect has been established, which can make switching to a stronger opioid necessary. Tramadol is considered a second-line analgesic. It can be used to treat nociceptive and neuropathic pain.

When treating strong nociceptive pain, μ‑opioid receptor agonists like hydromorphone, oxycodone, fentanyl, buprenorphine, and morphine are particularly suitable. Neuropathic pain can be treated using oxycodone, buprenorphine or morphine.

Tapentadol with its dual mechanism of action as an agonist of the μ‑opioid receptor and as a norepinephrine reuptake inhibitor (NRI) has also proven to be an efficient and safe analgesic option, in particular in the treatment of neuropathic pain [89].

When using opioids, it is paramount to take hepatic and renal comorbidities into account (Table 2). Tramadol, and less frequently, oxycodone and fentanyl, can cause a serotonin syndrome when combined with an existing therapy of monoamine oxidase inhibitors (MAO) inhibitors or SSRI, serotonin-noradrenalin-reuptake-inhibitor (SNRI), tricyclic antidepressants, mirtazapine or trazodone and triptans. 5‑HT3 antagonists in combination with tramadol reduce its analgesic efficacy.

**Table 2:** Analgesics used in cases of hepatic and/or renal insufficiency [104]

Drug name	Severe hepatic insufficiency	Renal insufficiency (glomerular filtration rate <30 ml/min)
NSAID, coxibs	Contraindicated	Contraindicated
Paracetamol	Is metabolised by enzymes in the liver into hepatotoxic N‑acetyl-p-benzoquinone imine and therefore contraindicated in case of liver damage	Longer intervals of 8–12 h
Dipyrone	No dosage adjustment	Dosage reduction
Duloxetine	Contraindicated	Contraindicated
Venlafaxine	50% dosage reduction	50% dosage reduction
Amitriptyline	CAUTION increased spasticity, dosage adjustment necessary	Dosage adjustment because of increased risk of urine retention
Gabapentin	No dosage adjustment	150–600 mg (divided into 3 daily doses)
Pregabalin	No dosage adjustment	25–150 mg (divided into 2 daily doses)
Tramadol	Longer dosage interval of 12 h necessary, maximum daily dose: 200 mg	Longer dosage interval of 12 h, maximum daily dose: 200 mg
Oxycodone	50% dosage reduction	50% dosage reduction
Hydromorphone	No dosage adjustment	No dosage adjustment
Fentanyl	No dosage adjustment	25% dosage reduction
Morphine	Longer dosage interval	25–50% dosage reduction
Buprenorphine	Possible dosage reduction	No dosage adjustment

*NSAID* nonsteroidal anti-inflammatory drugs

#### Adjuvants and co-analgesics

##### Antidepressants and anticonvulsants

As part of multimodal pain therapy, antidepressants generally play an important role in neuropathic pain treatment. Selective serotonin reuptake inhibitors (SSRI) such as citalopram, paroxetine, or escitalopram are to be used with caution in hemophilia patients as they can inhibit platelet aggregation and therefore increase the risk of bleeding. First-line therapy of neuropathic pain uses tricyclic antidepressants or serotonin-norepinephrine reuptake inhibitors (SNRI) that do not influence coagulation [8]. Using antidepressants to treat nociceptive pain is inadvisable. In addition, the dosage must always be strictly adapted to kidney and liver function. In cases of psychological changes in teenage pain patients, consulting psychological or psychiatric expertise is recommended.

Anticonvulsants such as gabapentin and pregabalin constitute first-line medication for the treatment of neuropathic pain. Adverse effects in the CNS include sedation, nausea, vomiting, headaches, dizziness, ataxia, and impaired vision; dermatoses like leukocytopenia and thrombocytopenia.

Dosage of both gabapentin and pregabalin must be adapted to kidney function.

##### Cannabinoids

For the treatment of acute pain, cannabinoids are not a suitable option but can have hyperalgesic effect. As for the treatment of chronic pain, limited data are available, making an evaluation difficult at this stage. Cannabinoids, both the psychoactive tetrahydrocannabinol (THC) and cannabidiol (CBD) which does not have psychoactive effects, can be used to treat neuropathic pain if first and second-line therapeutics show insufficient results. Cannabinoids generally only show a moderate analgesic effect of 30% on average. They can, however, have a positive effect on sleep, mood, and coping [90].

##### Glucocorticoids

Glucocorticoids have an antiphlogistic, anti-edematous, and analgesic effect, whereby the analgesia is brought on exclusively by the antiphlogistic properties.

At the outset of the therapy a high enough dosage is crucial; later on, however, need and necessity should be constantly monitored and the dosage reduced as quickly as possible. Patients need to be monitored closely in case of comorbidities, such as diabetes, cardiovascular diseases, peptic ulcer, recurring infection or glaucoma [62].

##### Hyaluronic acid, chondroitin sulphate, glucosamine

Cartilage tissue itself is not innervated. Consequently, the analgesic effects of hyaluronic acid, chondroitin sulphate, and glucosamine do not directly affect this tissue. Hyaluronic acid and its building blocks, chondroitin sulphate and glucosamine, can only ever be used to help rebuild worn, damaged, or lost cartilage. Intra-articular injections, however, should not be administered to hemophilia patients [91, 92].

Chondroitin sulphate can be used to treat arthrosis and is administered perorally. Studies and meta-analyses of the analgesic and function-enhancing effects of chondroitin sulphate reflect a conflicting data situation. Currently, there is no certain proof for a chondroprotective effect in arthrosis. A combination of orally administered chondroitin sulphate and glucosamine sulphate used to treat arthrosis of the knee has not produced better results than the placebo when it comes to pain symptoms and functionality [93].

Glucosamine is attributed with analgesic and anti-inflammatory effects and to protect or build up cartilage. At this time, however, there is no clear proof of any chondroprotective effects that glucosamine might have when used to treat arthrosis. A meta-analysis of orally administered glucosamine and chondroitin for arthritis showed that while chondroitin eased pain and improved functionality, glucosamine only improved joint mobility [94]. When used to treat osteoarthritis, glucosamine and chondroitin showed no reduction in pain or joint space narrowing compared to the placebo [95].

##### Phytopharmaceuticals

Although there is mixed evidence regarding their efficacy, a number of herbal agents are eligible for pain therapy [96]. According to a Cochrane review of arthrosis of the hip and knee, the use of plant-based medicines is neither recommended nor advised against [97]. There is, however, a body of sufficiently good evidence for a number of phytopharmaceuticals such as rampion or devil’s claw (*Harpagophytum procumbens*), willow bark extract, frankincense (*Boswellia serrata*) or comfrey root/herb (*Symphyti radix/herba*) and their analgesic/antiphlogistic effects for them to be rated as well-established use or at least traditional use. Their use allows for a reduction in conventional analgesics. Also, their tolerability is usually very good, which is probably a result of their multitarget effect [97–102].

Generally, the possibility of side effects and interactions in phytotherapy should not be underestimated. Potential interactions of phytopharmaceuticals that affect blood coagulation have to be considered: garlic and gingko biloba can reduce platelet aggregation when taken at the same time as anticoagulants, ginseng can reduce the effect of anticoagulants [103].

#### Polypharmacy and organ insufficiencies

When treating multimorbid patients it is paramount to only use medication that will not cause any strong side effects, especially in patients with limited kidney and liver function. In severe cases hepatic insufficiency can have a secondary effect on kidney function. There are numerous drugs for which the dosage has to be adapted to kidney and liver function (see Table 2; [104]).

With increasing comorbidities comes increasing polypharmacy which can make the choice of suitable analgesics significantly more difficult because the risk of interactions that may cause adverse effects increases. Selected interactions of analgesics are summarised in Table 1.

### Orthopaedic interventions and surgery in hemophilia

In spite of the fact that adequate and continuous pain therapy can often delay surgical procedures, they are frequently indispensable in hemophilia patients. Joint-preserving surgeries are used, such as radiosynoviorthesis, arthroscopic synovectomy or endoprosthetic care as well as non-joint-preserving methods, such as stiffening operations. Due to the increased risk of bleeding, infiltration therapy with cortisone is not an option for hemophilia patients.

#### Radiosynoviorthesis

Radiosynoviorthesis is a procedure which consists of injecting a short-acting radionucleid into the affected joint. This promotes sclerosis of the vessels and the hyperemic synovial tissue. This reduces hypertrophy [105]. Radiosynoviorthesis also significantly reduces bleeding and there even are indications that the progression of arthrosis may be slowed down [106].

#### Arthroscopic synovectomy

Due to osteoarthritis, hemophilia patients of an advanced adult age often require surgical interventions like tendon extension, synovectomy or joint replacement [107].

Before an arthroscopic synovectomy can be considered an MRI is necessary to assess cartilage condition, since patients without cartilage defects can profit from this measure the most. In a study on young patients, arthroscopic synovectomy led to a reduction of joint bleeds and pain and improved joint function. This significant improvement was still visible 6 years later [108]. The procedure did not influence the osteoarthritis stage, but endoprosthetic care could be delayed, which is a relevant aspect especially in young patients. The procedure is not indicated for advanced osteoarthritis with degenerative joint pain.

#### Endoprosthetic care

The advantages of endoprosthetic care are significant pain reduction as well as conservation or recovery of mobility. Disadvantages of this method include limited durability, which is particularly problematic in young active patients, an increased infection risk, and the necessity of several revision surgeries in the course of a patient’s life.

##### Knee joint endoprosthesis

In cases where the range of motion is limited to begin with, a total knee arthroplasty (TKA) can often not restore it completely. In hemophilia patients, implants are generally less durable than in non-hemophilia patients [109]. In many cases, however, the range of motion can be restored to at least twice the previous level. Significant functional improvement (Hospital for Special Surgery [HSS] score) can also be achieved, even if hemophilia patients have a higher risk of endoprosthetic failure [110].

##### Elbow joint endoprosthesis

Due to new, axially supported prosthetic designs, total elbow arthroplasties (TEA) are increasingly common. A follow-up survey showed significant improvement of the functional score but also high risk of infection and high rate of complications [111].

##### Ankle joint

Total ankle arthroplasties (TAA) are special insofar as the range of motion that can be reached with an artificial joint is very restricted; the limited anchoring in the bone is an additional disadvantage. As a consequence, arthrodesis is preferable to an endoprosthetic ankle joint replacement [112].

## Particularities in children and adolescents

Painful experiences because of hemophilia-related bleeds and treatment procedures start during early childhood. Psychological and physical experiences in this context can lead to negative conditioning in children as well as a further sensitisation to pain. Children have a lower pain threshold, and the pain perception and its verbalisation strongly depend on a child’s age. An interdisciplinary expert panel developed recommendations to improve pain management in children and adolescents with hemophilia [3].

The main particularities when it comes to measuring and treating pain in children with hemophilia will therefore only be briefly summarised here.

Children’s pain can be measured using a variety of tools such as the Childhood discomfort and pain scale (KUSS, Kindliche Unbehagens- und Schmerz Skala) [113] or the faces pain scale [18, 114]. For children with cognitive impairments the r‑FLACC scale (revised Face, Legs, Activity, Cry, Consolability) has proven particularly useful.

When it comes to subjecting children to necessary therapeutic measures, it is most important to show affection, give them a feeling of security, create a child-friendly environment, and provide distraction.

The effectiveness of psychological intervention as part of the long-term care for children with painful illnesses has been proven [115]. Psychological intervention is indicated especially in cases where the illness has a strong negative impact on daily life or when there are risk factors for chronification [116].

Children can already develop deficits in coordination, flexibility, strength ratio and endurance performance [36]. Therefore, a multimodal training is important [8, 36]. Physical measures like the additional use of laser therapy [66] or pulsed electromagnetic field therapy [65] play an important role to improve pain and physical function.

Pharmacological pain therapy for children is characterised by particularities in terms of pharmacodynamics and pharmacokinetics. Due to the developing hemostatic system, differences in pharmacodynamics are pronounced in the first 6 months of life but persist until puberty (Tanner II). In general, children have a more active metabolism and therefore often require a comparatively higher dosage per kg of body weight compared to adults to achieve a comparable therapeutic effect.

The types of drugs used on children beyond the first year of age include non-opioid analgesics like NSAIDs, dipyrone (metamizole), paracetamol, and opioids. The highest evidence level for the safe use in children is available for the NSAID ibuprofen which can be given rectally or orally but of note also impairs platelet function. Current results indicate that combinations of NSAIDs and paracetamol do not lead to better results than individual doses. Giving COX‑2 inhibitors is an option, but the data situation is insufficient for the use on children <12 years. Dipryone is recommended for pain therapy in children as long as special information and contraindications are taken into consideration [117, 118]. Antidepressants are also used effectively on school-age children and teenagers [119] and anticonvulsants such as gabapentin or pregabalin are among the first-line drugs used to treat neuropathic pain [119].

When non-opioid analgesics fail to produce the desired analgesic effect, opioids are used to supplement pain therapy in children of all ages. Interested readers are referred to the recommendations by Stromer et al. (in German) [3].

## Summary of recommendations

- Prophylactic treatment either with replacement of factor VIII or IX or non-factor therapies prevents bleedings, pain, and protects joint function. Beyond that, targeted pain therapy can be necessary.
- Physical and rehabilitative measures are used to complement therapy to treat acute pain and to prevent and treat chronic hemophilic arthropathy and the pain it causes. Physiotherapy and adequate pain therapy should be organised to go hand in hand. During acute phases the RICE principle (Rest, Ice, Compression, Elevation) is used, mobilisation should follow as soon as possible; however, in order to be able to initiate physical measures, adequate pharmacological analgesia is paramount and sufficient factor protection is equally important.
- Systematic analgesic therapy at an early stage can effectively ease pain, following the concepts of modern pain medicine, but adapted to the specific situation of hemophilia patients.
- Pain treatment should always focus on the underlying mechanisms of pain and not just its intensity; it should follow a multimodal approach and focus on the patient’s overall psychosocial status.
- Because of its effect on coagulation, acetylsalicylic acid is contraindicated for the treatment of hemophilia patients. If an NSAID is chosen, the substance’s affinity to COX‑1 should be as low as possible. Coxibs (selective COX‑2 inhibitors) constitute a valid alternative.
- Paracetamol (acetaminophen) is indicated for low to medium intensity of pain. Due to its low analgesic potency, paracetamol should only be used if a patient’s specific constellation does not allow for coxibs or the analgesically more potent non-opioid analgesic dipyrone (metamizole). In order to safely administer paracetamol, the patient’s age, body weight, duration of therapy, maximum daily dosage, and dosage intervals need to be taken into account.
- Compared to paracetamol (acetaminophen), dipyrone (metamizole) has a relatively high analgesic potency and a low potential for interactions and adverse effects.
- A combination of two non-opioids with a different mode of action, such as a coxib and dipyrone (metamizole), leads to a potentiation of the analgesic efficacy, therefore allowing for an up to 40% reduction in opioid dosage.
- If non-opioids fail to reach the desired analgesic effect or if pain intensity is high, the therapy should be complemented with opioids as soon as possible during an acute phase.
- When non-opioids in the case of chronic pain do not produce the desired analgesic effect, weak and strong opioids should be used complementarily. The choice of opioid depends on the pain category, the intensity of the pain, the character and rhythm of the pain, the receptors involved as well as possible contraindications.
- Adequate dosage and appropriate intervals are important to ensure almost complete pain reduction.
- Pain specialists should—insofar as they are available—be consulted when there are indications of pain chronification risk or when the treatment does not permit sufficient pain control.
- Since hemophilia patients are in constant contact with hemophilia centres, good interdisciplinary cooperation within the hospitals with hemophilia centres facilitates cooperation between hemophilia specialists, pain therapists, psychologists, and specialists for physical medicine as well as psychotherapists.
